# Niclosamide shows strong antiviral activity in a human airway model of SARS-CoV-2 infection and a conserved potency against the Alpha (B.1.1.7), Beta (B.1.351) and Delta variant (B.1.617.2)

**DOI:** 10.1371/journal.pone.0260958

**Published:** 2021-12-02

**Authors:** Anne Weiss, Franck Touret, Cecile Baronti, Magali Gilles, Bruno Hoen, Antoine Nougairède, Xavier de Lamballerie, Morten O. A. Sommer

**Affiliations:** 1 UNION therapeutics Research Services, Hellerup, Denmark; 2 Novo Nordisk Center for Biosustainability, Technical University Denmark, Lyngby, Denmark; 3 Unité des Virus Émergents (UVE: Aix-Marseille University -IRD 190-Inserm 1207-IHU Méditerranée, Infection), Marseille, France; 4 Institute Pasteur, Paris, France; 5 UNION Therapeutics, Hellerup, Denmark; University of Washington, UNITED STATES

## Abstract

SARS-CoV-2 variants are emerging with potential increased transmissibility highlighting the great unmet medical need for new therapies. Niclosamide is a potent anti-SARS-CoV-2 agent that has advanced in clinical development. We validate the potent antiviral efficacy of niclosamide in a SARS-CoV-2 human airway model. Furthermore, niclosamide remains its potency against the D614G, Alpha (B.1.1.7), Beta (B.1.351), and Delta (B.1.617.2) variants. Our data further support the potent anti-SARS-CoV-2 properties of niclosamide and highlights its great potential as a therapeutic agent for COVID-19.

## Introduction

Since its emerge in 2019, coronavirus disease 2019 (COVID-19) caused by severe acute respiratory syndrome coronavirus 2 (SARS-CoV-2) led to over 3.8 million deaths worldwide as of June 18^th^, 2021 [1]. A tremendous joint research effort led to the approval of several vaccines at unprecedented speed, yet anti-viral treatment options remain limited. At the same time, several viral variants harboring mutations in the N-terminal (NTD) and receptor-binding domain (RBD) of the spike protein gene, such as the B.1.1.7 (Alpha or 20I/501Y.V1), B.1.351 (Beta or 20H/501Y.V2), B.1.617.2 (Delta) are causing global concern as they have been associated with enhanced transmissibility and possible resistance to vaccines and antibody neutralization [2–7]. The B.1.1.7 and B.1.351 lineages have been linked to increased transmission of SARS-CoV-2 infection and B.1.617.2 to increased transmissibility and secondary attack rate [7–10]. The vaccine efficacy of ChAdOx1 nCoV-19 has been reported to be reduced to 10.4% against the B.1.351 variant [6]. Thus, despite the recent vaccine roll-out, there remains a high unmet need for novel therapeutics against SARS-CoV-2, which should be effective against circulating and potentially emerging variants of concern of SARS-CoV-2.

Niclosamide has been identified as a potent inhibitor of SARS-CoV-2 *in vitro* and *in vivo* and its optimized formulation for intranasal application and inhalation was well-tolerated in healthy volunteers in a Phase 1 trial [11–14]. Herein, we sought to further characterize the anti-viral properties of niclosamide by determining its potency in a human epithelial airway model of SARS-CoV-2 infection and tested its efficacy against several variants of concern of SARS-CoV-2.

## Material and methods

### Antiviral assay using human airway epithelia (HAE)

The effect of niclosamide on the replication of SARS-CoV-2 in the HAE bronchial model (Eptihelix) was determined as previously described by Touret *et al*. [15] and Pizzorno *et al*. [16]. Mucilair™ HAE reconstituted from human primary cells of bronchial biopsies from two different donors were purchased from Epithelix SARL (Geneva, Switzerland) and maintained in an air-liquid interface with specific media from Epithelix. The donor 1 epithelium was derived from a 56-year-old donor caucasian female with no existing pathologies reported or detected. The donor 2 epithelium was derived from a 17 year old male without any pathology reported. All samples provided by Epithelix SARL have been obtained with informed consent as part of studies or other processes that have had ethical review and approval. These studies were conducted according to the declaration of Helsinki on biomedical research (Hong Kong amendment, 1989), and received approval from local ethics commission (our study did not involve human participants and thus this statement is only applicable to Epithelix SARL regarding their commercial activity and is not relevant for our study that is only *in vitro*).

Briefly, human bronchial epithelial cells were apically infected with the European D614G strain of SARS-CoV-2 (BavPat1 D614G) at a multiplicity of infection (MOI) of 0.1 and cultivated in basolateral media that contained different concentrations of niclosamide ethanolamine (in duplicates) or no drug (virus control) for up to 4 days (treatment pre-infection and once daily post-infection). Media was renewed daily containing fresh niclosamide ethanolamine. Remdesivir was used as experimental positive control and non-treated samples as negative control. On day 2, 3 and 4 post-infection, samples were collected at the apical side and the viral titer was estimated with a TCID_50_ assay. On day 4, cells were lysed, and the intracellular viral RNA was extracted and quantified by qRT-PCR as described in the “IC50 determination” section. Statistical analysis was performed using the Ordinary Two-way Anova for TCID50 and Ordinary One-Way Anova for intracellular RNA, with Dunnett’s multiple comparisons test. Each treatment group was compared with its respective negative control of the same assay.

For the evaluation of cytotoxicity in HAE, LDH (lactate dehydrogenase) release was measured in the basal medium using the Cytotoxicity Detection KitPLUS (LDH) from Roche according to the manufacturer instructions on day 4 post-treatment in non-infected cells of donor 2. Assessing cytotoxicity based on LDH which is released from the cytosol of damaged cells has been previously performed in the context of HAE [17]. Each media from each assay duplicate was analysed minimum in duplicates. Statistical analysis was performed using the Ordinary One-way Anova with Dunnett’s multiple comparisons test. Raw data can be found in S1 Table.

### Cell lines

VeroE6 (ATCC CRL-1586) and Caco-2 (ATCC HTB-37) cells were grown in minimal essential medium (Life Technologies) with 7 .5% heat-inactivated fetal calf serum (FCS; Life Technologies), at 37°C with 5% CO_2_ with 1% penicillin/streptomycin (PS, 5000U.mL^−1^ and 5000μg.mL^−1^ respectively; Life Technologies), supplemented with 1 % non-essential amino acids (Life Technologies) and L-Glutamine (Life Technologies). VeroE6 TMPRSS2 cells (ID 100978) were obtained from CFAR and were grown in the same medium with the addition of G-418 (Life Technologies).

### Virus strains

SARS-CoV-2 strain BavPat1 was obtained from Pr. C. Drosten through EVA GLOBAL (https://www.european-virus-archive.com/). SARS-CoV-2 201/501YV.1 (Alpha) was isolated from a 18 years-old patient. The full genome sequence has been deposited on GISAID: EPI_ISL_918165. The strain is available through EVA GLOBAL: UVE/SARS-CoV-2/2021/FR/7b (lineage B 1. 1 .7, Alpha). SARS-CoV-2 Wuhan D614 strain was generated by ISA method. It contains the original D614 residue on the Spike protein. The strain is available through EVA GLOBAL UVE/SARS-CoV2/2020/FR/ISA_D614. SARS-CoV-2 SA (lineage B 1.351, Beta) was isolated in France in 2021 and is available through EVA GLOBAL: UVE/SARS-CoV-2/2021/FR/1299-ex SA (lineage B 1.351). B.1.617.2 (Delta; hCoV-19/France/PAC-0610/2021) was isolated in France in 2021 from an 87 old female patient and its full genome is deposited on GISAID: EPI_ISL_2838050.

To prepare the virus working stock, a 25cm^2^ culture flask of confluent VeroE6 cells growing with MEM medium with 2.5% FCS was inoculated at a MOI of 0.001. Cell supernatant medium was harvested at the peak of replication and supplemented with 25mM HEPES (Sigma-Aldrich) before being stored frozen in aliquots at -80°C. All experiments with infectious virus were conducted in a biosafety level 3 laboratory.

### IC50 and CC50 determination

One day prior to infection, 5×10^4^ VeroE6 / VeroE6 TMPRSS2 or 7×10^4^ Caco-2 cells per well were seeded in 100μL assay medium (containing 2.5% FCS) in 96 well culture plates. The next day, eight 2-fold serial dilutions of niclosamide ethanolamine were added to the cells in triplicate (25μL/well, in assay medium). Four virus control wells were supplemented with 25μL of assay medium. After 15 min, 25μL of a virus mix diluted in medium was added to the wells. The amount of virus working stock used was calibrated prior to the assay, based on a replication kinetics, so that the viral replication was still in the exponential growth phase for the readout as previously described [18, 19]. Four cell control wells (i.e. with no virus) were supplemented with 50μL of assay medium. On each culture plate, a control compound (Remdesivir, BLDpharm) was added in duplicate as experimental control. Plates were incubated for 2 days at 37°C prior to quantification of the viral genome by real-time RT-PCR. To do so, 100μL of viral supernatant was collected in S-Block (Qiagen) previously loaded with VXL lysis buffer containing proteinase K and RNA carrier. RNA extraction was performed using the Qiacube HT automat and the QIAamp 96 DNA kit HT following manufacturer instructions. Viral RNA was quantified by real-time RT-qPCR (GoTaq 1-step qRt-PCR, Promega) using 3.8μL of extracted RNA and 6.2μL of RT-qPCR mix and standard fast cycling parameters, *i*.*e*., 10min at 50°C, 2 min at 95°C, and 40 amplification cycles (95°C for 3 sec followed by 30sec at 60°C). Quantification was provided by four 2 log serial dilutions of an appropriate T7-generated synthetic RNA standard of known quantities (10^2^ to 10^8^ copies/reaction). RT-qPCR reactions were performed on QuantStudio 12K Flex Real-Time PCR System (Applied Biosystems) and analyzed using QuantStudio 12K Flex Applied Biosystems software v1.2.3. Primers and probe sequences, which target SARS-CoV-2 N gene, were: Fw: GGCCGCAAATTGCACAAT; Rev: CCAATGCGCGACATTCC; Probe: FAM-CCCCCAGCGCTTCAGCGTTCT-BHQ1. Viral inhibition was calculated as follow: 100* (quantity mean VC- sample quantity)/ quantity mean VC. The 50% and 90% inhibitory concentrations (IC50, IC90; compound concentration required to inhibit viral RNA replication by 50% and 90%) were determined using logarithmic interpolation as previously described [18]. For the evaluation of the 50% cytotoxic concentrations (CC50), the same culture conditions as for the determination of the EC50 were used, without addition of the virus, and cell viability was measured using CellTiter Blue® (Promega) following manufacturer’s instructions. CC50 was determined using logarithmic interpolation [18]. All data obtained were analyzed using GraphPad Prism 7 software. Raw data can be found in S2 and S3 Tables.

## Results and discussion

We first assessed niclosamide’s antiviral activity and cytotoxicity in two common cell lines: VeroE6 which has been extensively used at the beginning of the pandemic for antiviral compound profiling in which SARS-CoV-2 enters through Spike-induced fusion in endosomes and Caco-2 cells which naturally harbor ACE2 as well as the serine protease TMPRSS2 in a similar level to Calu-3 cells and thus SARS-CoV-2 enters via TMPRSS2 fusion at the plasma membrane [20]. We confirmed the antiviral potency shown in previous studies by Jeon *et al*. and Gassen *et al*. and found a similar IC_50_ (S1 Fig). [11, 12]. To strengthen the *in vitro* data with a preclinical model resembling the human respiratory tract, we employed a trans-well bronchial human airway epithelium (HAE) model infected with SARS-CoV-2. HAE cultured at an airway-liquid interface has been extensively used as an *in vitro* physiological model mimicking the human mucociliary airway epithelium to validate the effectivity of antivirals on infections in conducting airways [16, 21, 22].

**Fig 1:**
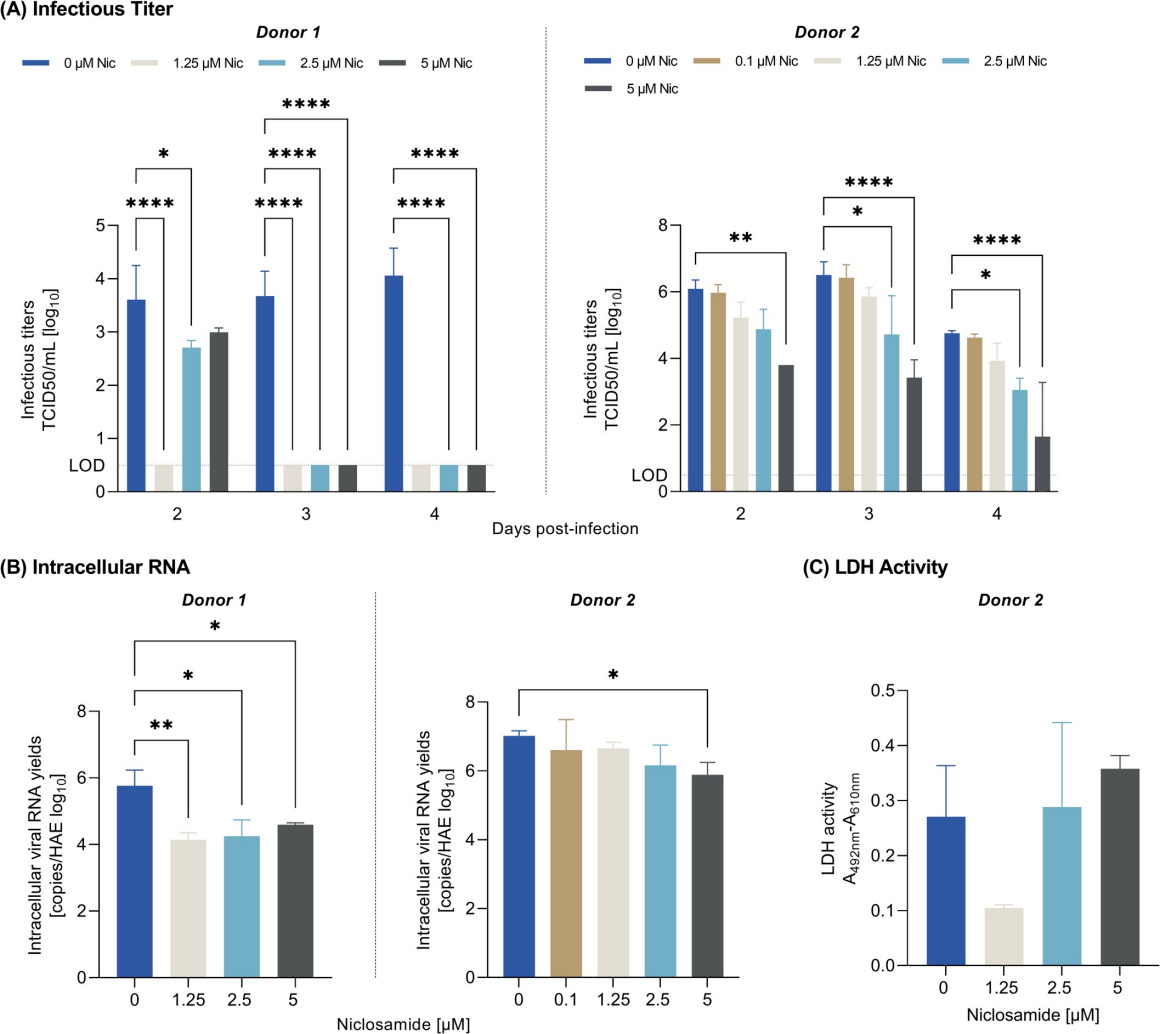
Antiviral efficacy of niclosamide in a trans-well model of human bronchial epithelium infected with SARS-CoV-2. Effect of niclosamide on the infectious titer on day 2, 3 and 4 (A) and intracellular viral RNA on day 4 (B) in two donors. (C) Effect of niclosamide on relative LDH activity as measure of cytotoxicity. **** = p < 0.0001, ** = p < 0.01, * = p < 0.05; Ordinary Two-Way ANOVA (A) and Ordinary One-Way ANOVA (B, C) with Dunnett’s multiple comparison test. Raw data underlying this figure are shown in S1 Table. Nic = Niclosamide.

Niclosamide exhibited strong anti-SARS-CoV-2 activity by reducing the infectious titer and intracellular RNA levels in the HAE model from two donors and three independent experiments. In donor 1, niclosamide treatment with concentrations ≥ 1.25 μM significantly reduced the infectious titer to below the limit of detection on day 3 and 4 (Fig 1A, left panel) and significantly reduced the intracellular viral RNA level on day 4 (Fig 1B, left panel). Aiming to determine the minimum effective concentration, a second independent experiment was performed with donor 1 cells using concentrations < 1.25 μM which confirmed the antiviral activity of niclosamide as 1 μM niclosamide significantly reduced the infectious titer on day 2–4 and the intracellular RNA level on day 4 (S2 Fig). In donor 2, ≥ 2.5 μM niclosamide significantly reduced the infectious titer reaching a maximum effect of a 3.1-log fold reduction (Fig 1A, right panel) and 5 μM niclosamide significantly reduced intracellular RNA levels on day 4 (Fig 1B, right panel). No cytotoxicity was observed with concentrations tested (effective concentrations used; 1.25–5 μM) as there was no significant increase in relative LDH activity on day 4 (Fig 1C).

Irrespective of the inter-donor variability in the degree of antiviral effect, these data validate the substantial anti-SARS-CoV-2 effect of niclosamide in a reconstituted human airway model.

We then tested the activity of niclosamide against several variants of concern of SARS-CoV-2, including the BavPat1 strain (D614G), SARS-CoV-2 lineage B.1.1.7 (Alpha), SARS-CoV-2 Wuhan D614, SARS CoV-2 lineage B.1.351 (Beta) and SARS-CoV-2 lineage B.1.617.2 (Delta). Since the Alpha variant has been shown to be attenuated on VeroE6 cells (e.g. low relative infectivity, lower virus titer, smaller plaque size) we used VeroE6 TMPRSS2 cells to test for antiviral efficacy against the variants of concerns [23].

**Fig 2:**
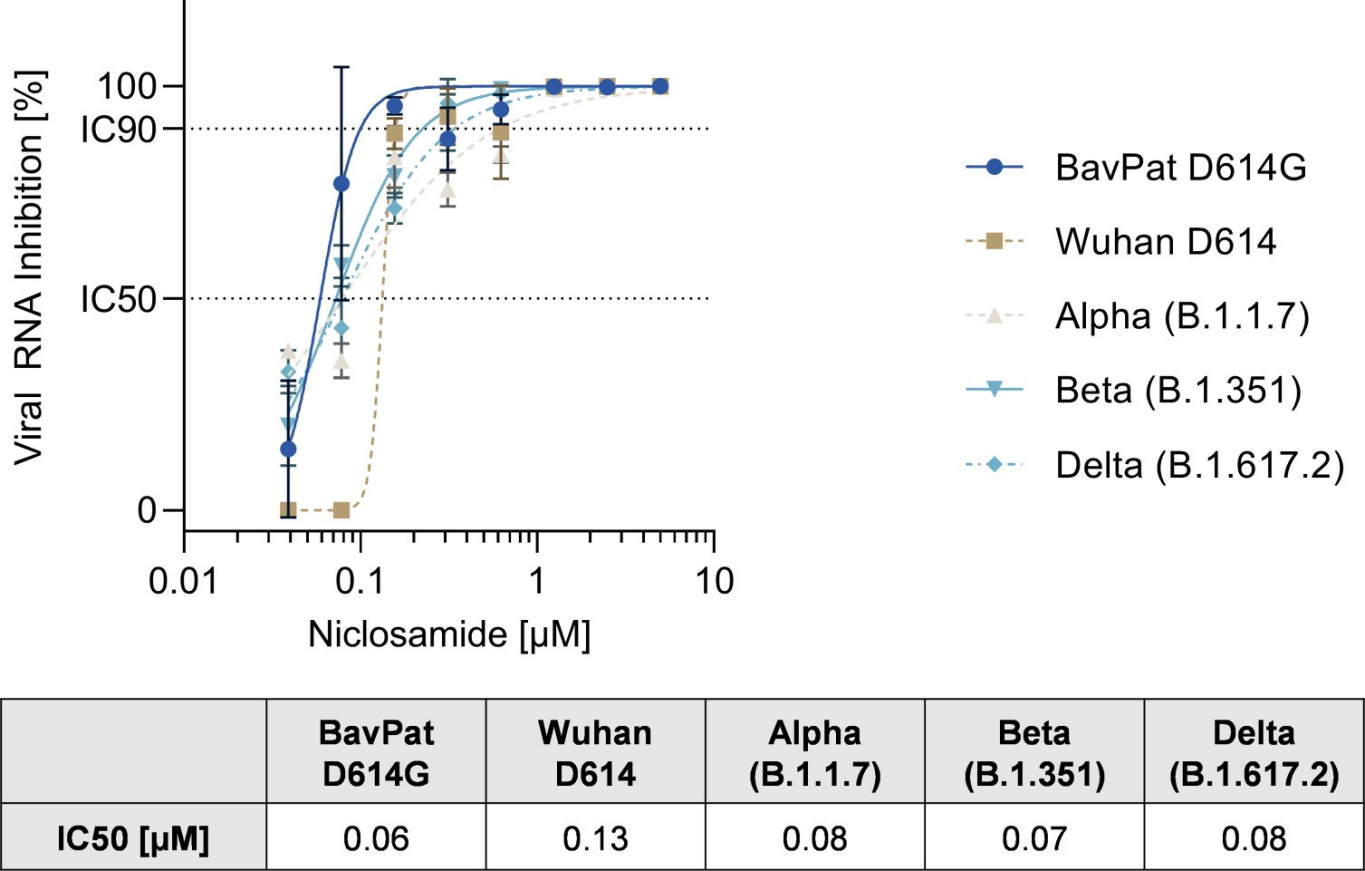
Effect of niclosamide on SARS-CoV-2 variants, including Alpha (B.1.1.7), Beta (B.1.351) and Delta (B.1.617.2) in VeroE6 TMPRSS2 cells. IC = Inhibitory concentration. N = 3. Raw data underlying this figure are shown in S2 Table.

Niclosamide inhibited replication of the SARS-CoV-2 original strain (Wuhan D614) in VeroE6 TMPRSS2 cells with an IC_50_ of 0.13μM and IC_90_ of 0.16 μM which is in accordance with previous studies [11, 12]. Importantly, niclosamide also blocked the replication of the European BavPat D614G, B.1.1.7, B.1.351 and B.1.617.2 variant with an IC_50_ of 0.06 μM, 0.08 μM, 0.07 μM, and 0.08 μM respectively (Fig 2).

Thus, niclosamide is effective against all tested variants of SARS-CoV-2 having a similar potency across the different strains compared to the original Wuhan D614 strain. These data are in accordance with the recently published study by Lee et al. demonstrating that niclosamide is equally effective against the Alpha and Beta variant in VeroE6 and Calu-3 cells [24].

Furthermore, these data are in line with the host-targeted mode of action of niclosamide, which has been described to interfere with basic cellular mechanisms involved in SARS-CoV-2 replication, such as autophagy, the endosomal pathway and the TMEM16A chloride channel [12, 25–27]. The molecule will also deserve further investigations to assess its potential role in the chemotherapeutic armamentarium required for future emerging infectious disease preparedness. Furthermore, the deployment of combination therapies with drugs that complement the mode of action of niclosamide warrants further evaluation as an effective tool in the combat of the current and a potential future pandemic.

Taken together, our findings support niclosamide’s therapeutic potential as a potent anti-viral agent against SARS-CoV-2, including its variants of concern. Trials in patients with COVID-19 are needed to substantiate future clinical use.

## Supporting information

S1 Fig Antiviral efficacy of niclosamide against SARS-CoV-2 (BavPat D614G) in VeroE6 and Caco-2 cells. Normalized response is displayed in (A) and raw fluorescence data in (B). N = 3. IC = inhibitory concentration. CC = cytotoxic concentration.
(TIF)

S2 Fig Minimum antiviral effective concentration of niclosamide in a trans-well model of human bronchial epithelium infected with SARS-CoV-2. Effect of niclosamide on the infectious titer on day 2, 3 and 4 (A) and intracellular viral RNA on day 4 (B) in donor 1. **** = p < 0.0001, *** = p < 0.001, * = p < 0.05; Ordinary Two-Way ANOVA (A) and Ordinary One-Way ANOVA (B) with Dunnett’s multiple comparison test. Raw data underlying this figure are shown in S1 Table. Nic = Niclosamide.
(TIF)

S1 Table Raw data underlying Figs 1 and S2. Concentration of niclosamide in μM.
(DOCX)

S2 Table Raw data underlying Fig 2. Data presented as viral RNA inhibition [%].
(DOCX)

S3 Table Raw data underlying S1 Fig. Data presented as % normalized to control or as raw data.
(DOCX)
