# Supplementary figures and images for: Niclosamide shows strong antiviral activity in a human airway model of SARS-CoV-2 infection and a conserved potency against the Alpha (B.1.1.7), Beta (B.1.351) and Delta variant (B.1.617.2)
Source: PLoS One. 2021 Dec 2;16(12):e0260958. doi: 10.1371/journal.pone.0260958 (PMC8639074; doi:10.1371/journal.pone.0260958)

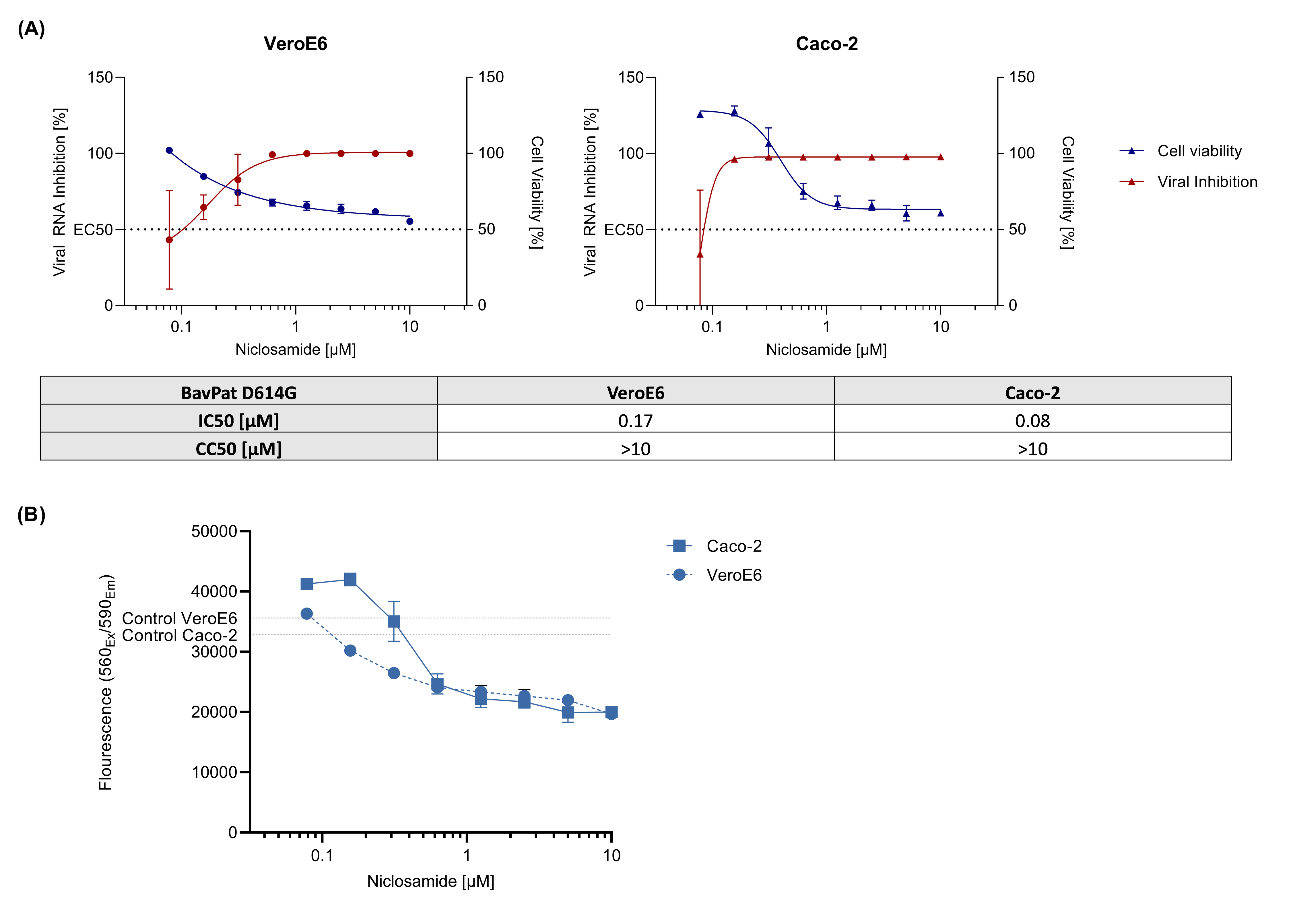

Supplement: S1 Fig — Normalized response is displayed in (A) and raw fluorescence data in (B). N = 3. IC = inhibitory concentration. CC = cytotoxic concentration. (TIF) [file pone.0260958.s001.tif]

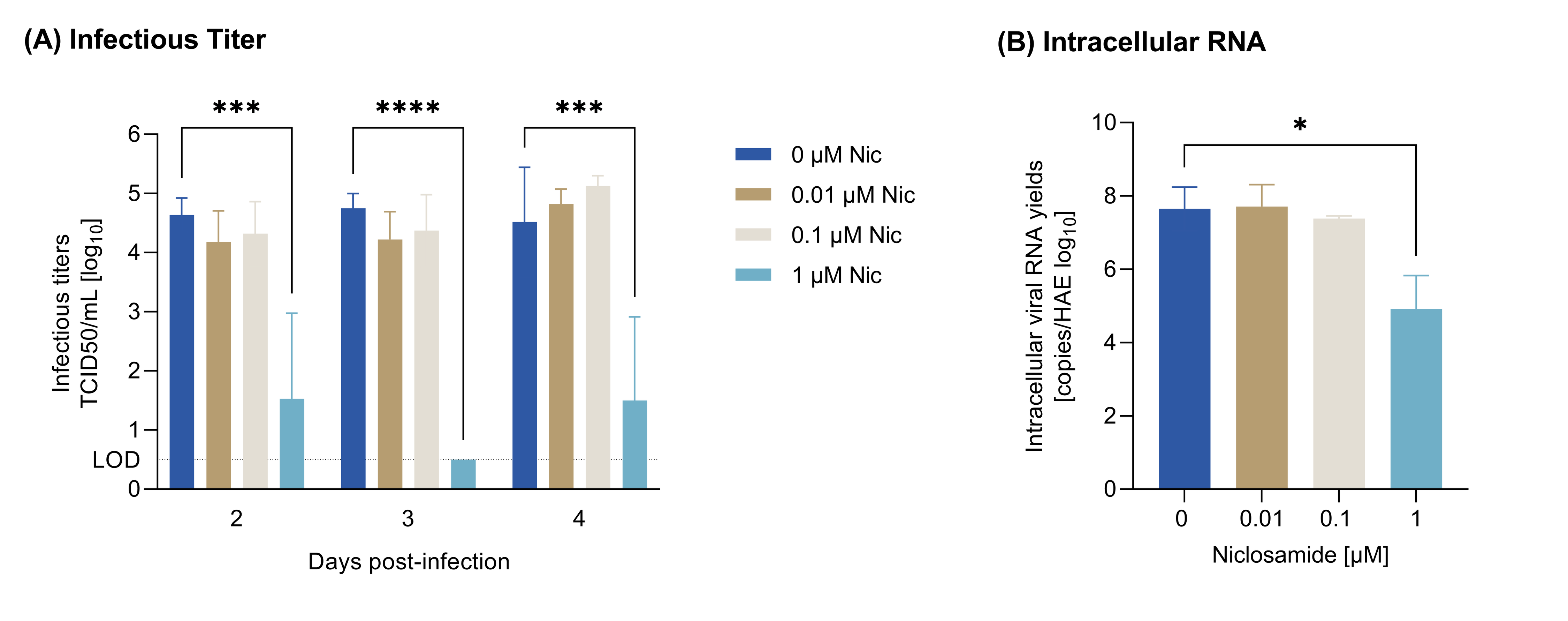

Supplement: S2 Fig — Effect of niclosamide on the infectious titer on day 2, 3 and 4 (A) and intracellular viral RNA on day 4 (B) in donor 1. **** = p < 0.0001, *** = p < 0.001, * = p < 0.05; Ordinary Two-Way ANOVA (A) and Ordinary One-Way ANOVA (B) with Dunnett’s multiple comparison test. Raw data underlying this figure are shown in S1 Table. Nic = Niclosamide. (TIF) [file pone.0260958.s002.tif]
