# Supplementary material for: Niclosamide shows strong antiviral activity in a human airway model of SARS-CoV-2 infection and a conserved potency against the Alpha (B.1.1.7), Beta (B.1.351) and Delta variant (B.1.617.2)
Source: PLoS One. 2021 Dec 2;16(12):e0260958. doi: 10.1371/journal.pone.0260958 (PMC8639074; doi:10.1371/journal.pone.0260958)
Supplement: S1 Table — Concentration of niclosamide in μM. (DOCX) [file pone.0260958.s003.docx]

**S1 Table. Raw data underlying Fig 1 and S2 Fig.** Concentration of niclosamide in µM.

| **Donor** | **Day** | **Infectious Titer TCID_50_/mL [log10]** | | | | | | | | | | | | | | | |
| --- | --- | --- | --- | --- | --- | --- | --- | --- | --- | --- | --- | --- | --- | --- | --- | --- | --- |
|  |  | **0** | | | | **1.25** | | **2.5** | | **5** | | | | | | | |
| 1 | 2 | 3.30103 | 3.051153 | 4.518421 | 3.551084 | 0.499687 | 0.499687 | 2.801061 | 2.611405 | 2.937418 | 3.051153 | | | | | | |
|  | 3 | 3.664642 | 3.176091 | 4.30103 | 3.551084 | 0.499687 | 0.499687 | 0.499687 | 0.499687 | 0.499687 | 0.499687 | | | | | | |
|  | 4 | 4.518422 | 3.456214 | 4.456202 | 3.801061 | 0.499962 | 0.499962 | 0.499962 | 0.499962 | 0.499962 | 0.499962 | | | | | | |
| **Donor** | **Day** | **0** | | | | **0.01** | | **0.1** | | **1** | | | | | | | |
| 1 | 2 | 4.801033 | 4.801033 | 4.30103 | | 4.55103502 | 3.80106053 | 4.70103234 | 3.93741758 | 2.55108387 | 0.49968708 | | | | | | |
|  | 3 | 4.901028 | 4.884365 | 4.456199 | | 4.55103502 | 3.88434215 | 4.80103306 | 3.93741758 | 0.49968708 | 0.49968708 | | | | | | |
|  | 4 | 5.3 | 4.75 | 3.5 | | 4.63636364 | 5.000 | 5.25 | 5 | 2.49996187 | 0.49968708 | | | | | | |
| **Donor** | **Day** | **0** | | | | **0.1** | | **1.25** | | **2.5** | | **5** | | | | | |
| 2 | 2 | 5.80103 | 6.17603 | 6.30103 | | 5.80103 | 6.14478 | 5.55103 | 4.90103 | 4.456202 | 5.30103 | 3.80103 | 3.80103 | | | | |
|  | 3 | 6.664666 | 6.80103 | 6.05103 | | 6.70103 | 6.14478 | 5.664666 | 6.05103 | 3.90103 | 5.544273 | 3.05103 | 3.80103 | | | | |
|  | 4 | 4.80103 | 4.80103 | 4.664666 | | 4.55103 | 4.70103 | 3.55103 | 4.30103 | 2.80103 | 3.30103 | 2.80103 | 0.499687 | | | | |
| **Donor** | **Day** | **Intracellular viral RNA yields [copies/HAE log10]** | | | | | | | | | | | | | | | |
|  |  | **0** | | | | **1.25** | | **2.5** | | **5** | | | | | | | |
| 1 | 4 | 5.836658 | 5.510171 | 6.389993 | 5.309994 | 4.28751 | 3.994992 | 4.593375 | 3.899164 | 4.558327 | 4.63573 | | | | | | |
| **Donor** | **Day** | **0** | | |  | **0.01** | | **0.1** | | **1** | | | | | | | |
| 1 | 4 | 6.994397 | 7.770192 | 8.167787 | | 7.28186206 | 8.12803941 | 7.32752753 | 7.43093476 | 4.25860742 | 5.55994245 | | | | | | |
| **Donor** | **Day** | **0** | | | | **0.1** | | **1.25** | | **2.5** | | **5** | | | | | |
| 2 | 4 | 7.141778 | 7.060584 | 6.85934 | | 5.974182 | 7.230633 | 6.77678 | 6.52517 | 5.741593 | 6.576198 | 6.142711 | 5.632608 | | | | |
| **Donor** | **Day** | **Relative LDH activity** | | | | | | | | | | | | | | | |
| 2 | 4 | **0** | | | | **1.25** | | | | **2.5** | | | | **5** | | | |
|  |  | 0.3682 | 0.331999 | 0.1846 | 0.1974 | 0.0982 | 0.1053 | 0.1048 | 0.1116 | 0.4286000 | 0.4136 | 0.1488 | 0.162 | 0.37380001 | 0.32229999 | 0.3635 | 0.37149999 |
