# Supplementary material for: Niclosamide shows strong antiviral activity in a human airway model of SARS-CoV-2 infection and a conserved potency against the Alpha (B.1.1.7), Beta (B.1.351) and Delta variant (B.1.617.2)
Source: PLoS One. 2021 Dec 2;16(12):e0260958. doi: 10.1371/journal.pone.0260958 (PMC8639074; doi:10.1371/journal.pone.0260958)
Supplement: S2 Table — Data presented as viral RNA inhibition [%]. (DOCX) [file pone.0260958.s004.docx]

**S2 Table. Raw Data underlying Fig 2.** Data presented as viral RNA inhibition [%].

| Niclosamide[µM] | BavPat D614G | | | Wuhan D614 | | | Alpha (B.1.1.7) | | | Beta (B.1.351) | | | Delta (B.1.617.2) | | |
| --- | --- | --- | --- | --- | --- | --- | --- | --- | --- | --- | --- | --- | --- | --- | --- |
| 5 | 99.98 | 99.97 | 99.99 | 100 | 100 | 99.95 | 99.86 | 99.96 | 99.86 | 100 | 100 | 100 | 99.96 | 99.75 | 99.59 |
| 2.5 | 99.99 | 99.88 | 99.56 | 99.97 | 100 | 100 | 99.78 | 99.89 | 99.8 | 100 | 99.97 | 99.96 | 99.9 | 99.54 | 99.91 |
| 1.25 | 99.92 | 99.89 | 99.88 | 99.98 | 99.81 | 99.98 | 98.93 | 99.7 | 99.68 | 99.52 | 99.83 | 99.96 | 98.58 | 99.42 | 99.62 |
| 0.625 | 95.23 | 97.7 | 90.83 | 94.95 | 96.02 | 76.41 | 86.04 | 82.48 | 83.26 | 98.92 | 99.59 | 99.5 | 97.96 | 97.9 | 99.02 |
| 0.3125 | 84.88 | 81.91 | 95.94 | 95.9 | 97.43 | 85.24 | 75.6 | 71.77 | 79.72 | 99.34 | 83.48 | 96.87 | 93.52 | 96.99 | 97.51 |
| 0.15625 | 94.96 | 93.55 | 97.51 | 92.35 | 85.11 | 89.06 | 90.24 | 84.02 | 75.76 | 77.15 | 84.31 | 74.61 | 73.61 | 73.11 | 67.08 |
| 0.078125 | 100 | 84.68 | 46.55 | 0 | 0 | 0 | 38.63 | 36.53 | 30.83 | 53.69 | 63.17 | 56.24 | 43.57 | 30.88 | 54.52 |
| 0.0390625 | 0 | 11.35 | 31.9 | 0 | 0 | 0 | 38.35 | 38.01 | 36.4 | 27.93 | 9.53 | 22.47 | 38.47 | 30.28 | 29.27 |
