# Supplementary material for: Niclosamide shows strong antiviral activity in a human airway model of SARS-CoV-2 infection and a conserved potency against the Alpha (B.1.1.7), Beta (B.1.351) and Delta variant (B.1.617.2)
Source: PLoS One. 2021 Dec 2;16(12):e0260958. doi: 10.1371/journal.pone.0260958 (PMC8639074; doi:10.1371/journal.pone.0260958)
Supplement: S3 Table — Data presented as % normalized to control or as raw data. (DOCX) [file pone.0260958.s005.docx]

**S3 Table. Raw Data underlying S1 Fig.** Data presented as % normalized to control or as raw data.

**(A) Data presented as % normalized response to control**

| Normalized Response % | Niclosamide [µM] | Vero E6 viral inhibition | | | Vero E6 viability | | | Caco-2 viral inhibition | | | Caco-2 viability | | |
| --- | --- | --- | --- | --- | --- | --- | --- | --- | --- | --- | --- | --- | --- |
|  | 10 | 99.97 | 99.98 | 99.98 | 55.93 | 54.63 | 55.25 | 97.65 | 98.76 | 97.12 | 60.18 | 62.52 | 60.24 |
|  | 5 | 99.97 | 99.97 | 99.97 | 62.98 | 62.88 | 59.21 | 97.77 | 97.79 | 97.82 | 65.79 | 60.72 | 55.70 |
|  | 2.5 | 99.98 | 99.97 | 99.98 | 67.09 | 62.09 | 61.69 | 97.41 | 98.36 | 97.36 | 68.93 | 66.83 | 62.56 |
|  | 1.25 | 99.96 | 99.95 | 99.96 | 68.89 | 64.23 | 63.41 | 97.77 | 97.69 | 97.54 | 71.26 | 68.91 | 62.87 |
|  | 0.625 | 99.36 | 99.02 | 99.33 | 70.38 | 66.55 | 66.23 | 98.12 | 98.01 | 97.08 | 80.04 | 75.89 | 69.79 |
|  | 0.3125 | 93.23 | 63.31 | 91.34 | 74.96 | 73.40 | 74.61 | 97.31 | 97.76 | 97.82 | 97.55 | 117.52 | 105.34 |
|  | 0.15625 | 65.69 | 72.02 | 55.89 | 86.81 | 82.82 | 84.71 | 95.53 | 97.13 | 96.25 | 128.89 | 130.57 | 124.37 |
|  | 0.078125 | 59.3 | 64.25 | 5.91 | 102.58 | 101.95 | 101.56 | 82.07 | 14.95 | 4.92 | 126.06 | 124.01 | 127.18 |

**(B) Data presented as raw data**

| Flourescence (560_Ex_/590_Em_) | Niclosamide [µM] | Vero E6 viability | | | Caco-2 viability | | |
| --- | --- | --- | --- | --- | --- | --- | --- |
|  | 10 | 19928 | 19462 | 19686 | 19757 | 20523 | 19775 |
|  | 5 | 22437 | 22401 | 21096 | 21596 | 19934 | 18284 |
|  | 2.5 | 23901 | 22120 | 21980 | 22628 | 21937 | 20536 |
|  | 1.25 | 24544 | 22885 | 22593 | 23392 | 22622 | 20638 |
|  | 0.625 | 25075 | 23709 | 23595 | 26275 | 24914 | 22910 |
|  | 0.3125 | 26706 | 26150 | 26583 | 32024 | 38577 | 34579 |
|  | 0.15625 | 30927 | 29508 | 30179 | 42312 | 42864 | 40828 |
|  | 0.078125 | 36546 | 36321 | 36184 | 41383 | 40709 | 41749 |
|  | 0 | 38330 |  |  | 37432 |  |  |
|  | 0 | 33665 |  |  | 33590 |  |  |
|  | 0 | 34630 |  |  | 31587 |  |  |
|  | 0 | 34607 |  |  | 32965 |  |  |
|  | 0 | 34726 |  |  | 29512 |  |  |
|  | 0 | 35343 |  |  | 32614 |  |  |
|  | 0 | 35660 |  |  | 32010 |  |  |
|  | 0 | 38060 |  |  | 32907 |  |  |
